# Dynamic visual effects enhance flower conspicuousness but compromise color perception

**DOI:** 10.1126/sciadv.adz9010

**Published:** 2025-11-26

**Authors:** Alexander Dietz, Johannes Spaethe, Casper J. van der Kooi

**Affiliations:** ^1^ Behavioral Physiology and Sociobiology, University of Würzburg, Würzburg, Germany.; ^2^ Groningen Institute for Evolutionary Life Sciences, University of Groningen, Groningen, Netherlands.

## Abstract

Dynamic visual effects such as glossiness are taxonomically widespread and have evolved repeatedly across the tree of life. Their changeable nature poses a challenge for reliable signaling, because for signals to be reliable, they should be consistent. Glossy visual effects defy that principle, because the bright, directional pulse of light that dominates their appearance is highly variable across space and time. Here, we investigate how dynamic light reflections influence signal efficacy using bumblebees as a model of insect vision and plant-pollinator interactions. We show that glossy floral signals occur across Angiosperm lineages. Through behavioral experiments with artificial stimuli that mimic the spectral and spatial reflectance properties of glossy and matte floral surfaces, we demonstrate that glossiness enhances long-range detectability but compromises fine-scale color discrimination at close range. Glossiness thus poses an optical property by which organisms can attune their visual appearance independent of pigmentary properties, representing a functional trade-off between conspicuousness and signal reliability.

## Teaser

Dynamic visual signaling has evolved in flowers to exploit perceptual constraints in pollinators.

## INTRODUCTION

The natural world is resplendent with visual effects that are used by animals and plants to communicate. Some of nature’s most vibrant visual signals are those of glossy structures. Examples include glossy leaves and flowers, glittering fish scales, mirror-like reflections of beetle carapaces, and lustrous visual effects emitted by the flapping wings of birds and butterflies (*1*–*8*). When these organisms move with respect to each other, their visual appearance is dominated by a flash: a highly directional and bright pulse of light. Why are some organisms glossy and others not?

The occurrence of glossy visual effects is paradoxical in two ways. First, the signaling structures of many terrestrial organisms are matte. When objects have a matte color, their visual appearance is consistent across time and space. Theory predicts that signal consistency enhances a signal’s reliability (*2*, *9*, *10*). However, visually dynamic, glossy surface coloration has evolved in a wide range of plants and animals, which suggests that glossiness can be beneficial. Second, glossy signals are expected to constitute a visual trade-off. On one hand, the flash effect emitted by glossy objects may enhance long-range visibility (*6*). This may be caused by the temporal component of the dynamicity (“on-off”) as well as because a bright pulse of light increases the distance at which photoreceptors can detect the object. On the other hand, specular, mirror-like reflections undermine an object’s chromatic signal (i.e., its color), because surface reflections are not modulated by pigments inside the flower, which may reduce short-range discriminability (*6*, *11*–*14*). Specular reflections may furthermore overwhelm or even blind the observer, when the high brightness overexposes the photoreceptors or the visual neurons and so impede object recognition. The impact of both the long- and short-range effects are expected to be particularly pronounced in animals with limited visual acuity, such as animals with compound eyes (*15*). For example, *Drosophila melanogaster* fruit flies avoid flying over glossy surfaces, because glossiness hampers their visual navigation (*16*).

We investigate the functional importance of glossiness for visual signaling using a plant-pollinator system. Analysis of uncommon but phylogenetically widespread glossy floral surfaces reveals that surface gloss has evolved repeatedly in unrelated Angiosperm lineages. To understand the impact of surface gloss on flower detection and discrimination by pollinators, we use bumblebees (*Bombus terrestris*), a model organism in visual ecology. We thus find that surface gloss conveys a visual ecological trade-off by enhancing long-distance detection but hampering short-distance color discrimination.

## RESULTS

### Glossy flowers are rare but widespread in Angiosperms

**Fig. 1.**
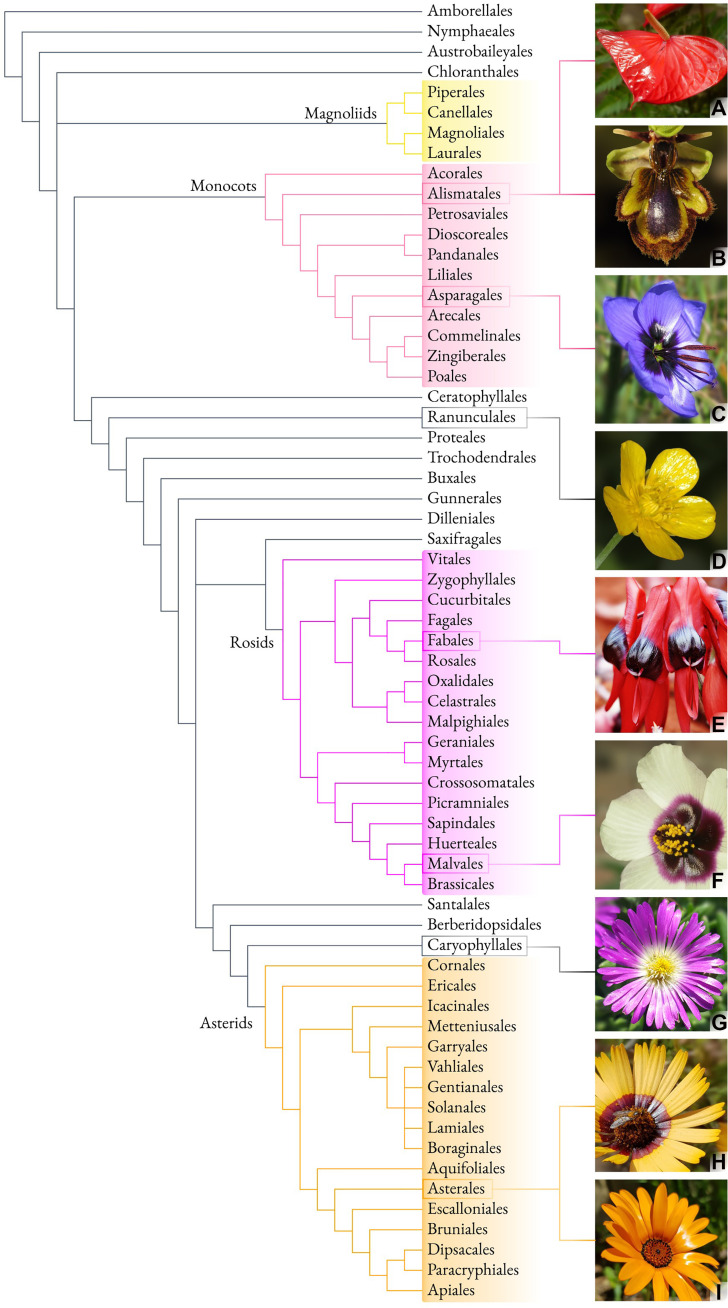
Examples of glossy flowers in Angiosperms. Species highlighted on the right: *Anthurium andraeanum* (**A**), *Ophrys speculum* (**B**), *Geissorhiza splendidissima* (**C**), *Ranunculus repens* (**D**), *Swainsona formosa* (**E**), *Hibiscus cannabinus* (**F**), *Delosperma* spp. (**G**), *Ursinia anthemoides* (**H**), *Dimorphoteca sinuata* (**I**). Picture credits: (A) K. Lunau; [(C), (H), and (I)]: A. Ellis; (E) Peter Firus/Flagstaffotos.

Glossy floral signaling structures are not the plesiomorphic condition in Angiosperms (*17*–*19*) but are phylogenetically widespread. Examples of flowers that are clearly glossy to the human eye can be found in buttercups, sexually deceptive orchids, South African daisies, succulents, and bird-pollinated Australian desert plants (Fig. 1). Many more species have been reported to display glossy floral signaling structures (*4*, *8*, *18*, *20*–*22*). Together, these examples suggest that floral gloss is not restricted to specific phylogenetic groups, pollinator types or pigmentation (*17*–*19*). The repeated occurrence of glossy floral signaling structures across the Angiosperm phylogeny supports the hypothesis of a potential selective advantage for visibility.

### Optical properties of glossy and matte flowers

**Fig. 2.**
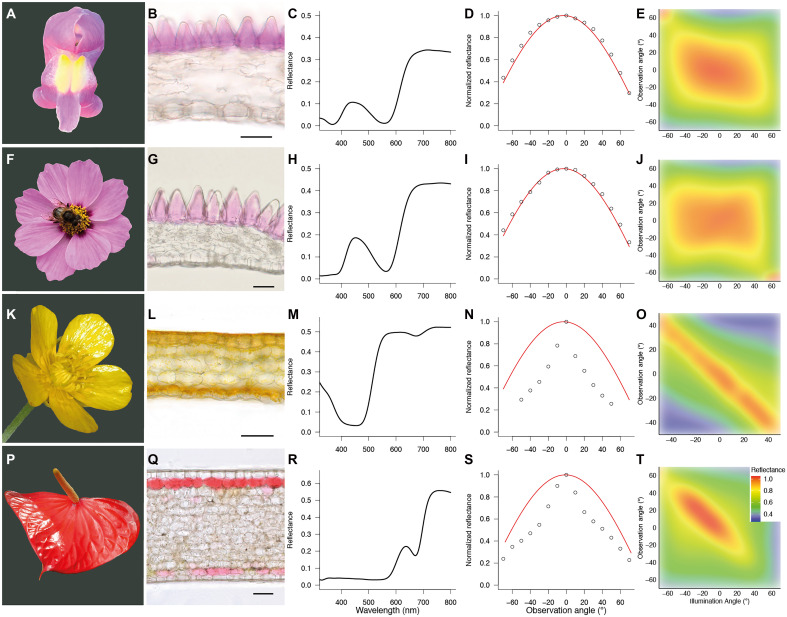
Optical and anatomical properties of glossy and matte flowers. The matte flowers of *A. majus* (**A**) and *C. bipinnatus* (**F**) and the glossy flowers of *Ranunculus repens* (**K**) and *Anthurium andraeanum* (**P**) are analyzed. Cross sections show the cone-shaped (**B** and **G**) or flat (**L** and **Q**) epidermal cells. Scale bars, 50 μm. Integrating sphere spectra are shown (**C**, **H**, **M**, and **R**). Normalized reflectance as a function of detection angle under perpendicular illumination, shown as open circles, follows a cosine fit (red curve) for cone-shaped surfaces (**D** and **I**) and clearly deviates from a cosine for glossy surfaces (**N** and **S**). Normalized reflectance as a function of angle of detection and angle of illumination reveals a spatially wide distribution for cone-shaped surfaces (**E** and **J**) and (imperfect) mirroring by glossy surfaces (**O** and **T**).

To analyze the optical properties of glossy and matte flowers, we quantified the spatial and spectral reflectance characteristics of several unrelated species. Floral surfaces of *Antirrhinum majus*, a model system for floral epidermal cell shape and pigmentation (*17*, *23*), are sculptured with conical epidermal cells of about 30 μm high (Fig. 2, A and B). Similar cone-shaped surfaces are found in *Cosmos bipinnatus* (Fig. 2, F and G) (*24*) and most other Angiosperms perianths (*17*–*19*). For cone-shaped floral epidermal cells, the reflectance as a function of observation angle follows a cosine (Fig. 2, D and I), meaning that the spatial reflectance function mimics that of a near-perfect, Lambertian diffuser (*25*). The spatially wide reflection pattern is further demonstrated by plotting reflectance as a function of angle of incidence and angle of observation (Fig. 2, E and J). By contrast, for flowers with flat epidermal cells, such as those found in *Ranunculus* (Fig. 2K, L), *Anthurium* (Fig. 2P, Q), and other glossy-flowered species (*4*, *18*, *20*), the reflectance as a function of observation angle clearly deviates from a cosine (Fig. 2, N and S). Reflectance is highest when the angles of illumination and observation are identical and opposite, i.e., in the mirror angle (Fig. 2, O and T). Glossy flowers are imperfect mirrors, because under mirroring angles, the high-reflectance band is ~10° to 20° wide (Fig. 2, O and T). This is caused by inhomogeneities in the flower local surface, such as veins (Fig. 2P), which somewhat enlarge the spatial spread of light reflected by the surface.

**Fig. 3.**
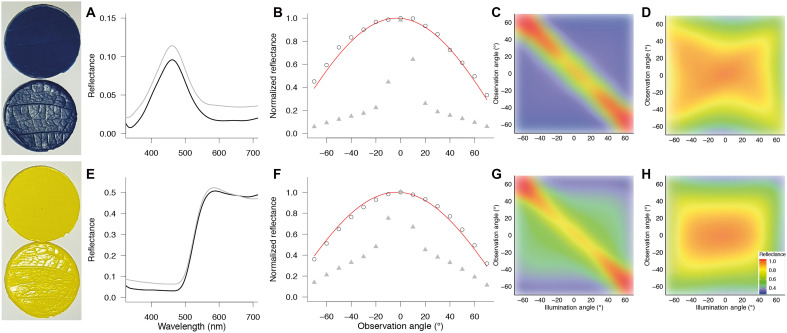
The optical properties of the artificial stimuli used for the behavioral experiments. Reflectance spectra obtained with an integrated sphere (**A** and **E**) show that reflectance is ~3% higher in glossy (gray lines) than matte stimuli (black lines). Under perpendicular illumination, reflectance as a function of observation angle follows a cosine (red curve) for matte stimuli (open circles) but not for glossy stimuli (**B** and **F**; gray triangles). The normalized reflectance as a function of detection and observation angle shows imperfect mirror-like reflection in glossy (**C** and **G**) and a spatially wide reflection pattern for matte stimuli (**D** and **H**).

It is unknown if insect pollinators perceive and respond to glossiness. To test the perception of surface gloss by bumblebees (*B. terrestris*), we developed two types of artificial epoxy stimuli that are identical in all aspects except surface reflection. The stimuli are facsimiles of cone-shaped or flat flower epidermal surfaces, and their spectral and spatial reflection characteristics mimic those of matte and glossy flowers (Fig. 2). The reflectance of stimuli with a flat surface measured with an integrating sphere is ~3% higher than for stimuli with a cone-shaped surface (Fig. 3, A and E). An about 3% reflectance difference is similar to the measured surface reflectance of glossy leaves and flowers as well as to calculated reflectance of an air-cuticle interface (*26*–*28*). For artificial stimuli with cone-shaped epidermal cells, reflectance as a function of detection angle follows a cosine, whereas for glossy stimuli, it clearly deviates from a cosine fit close to normal illumination (Fig. 3, B and F). The heatmaps further show the imperfect mirroring effect of glossy (Fig. 3, C and G) and spatially homogeneous reflection patterns of matte flowers (Fig. 3, D and H). To disentangle the effect of pigment-based color from surface reflection for visual signaling, artificial stimuli were colored by adding different pigments to the epoxy. Using combinations of blue or yellow pigments, we created pairs of stimuli with varying degrees of intrapair color and/or achromatic contrast, quantified through behaviorally validated bumblebee color spaces and long-wavelength photoreceptor contrasts (*29*–*32*). We used the artificial stimuli to test for bumblebee innate preference, impact on short-range object discrimination, and impact on long-range detection of surface gloss.

### A glossy visual ecological trade-off

**Fig. 4.**
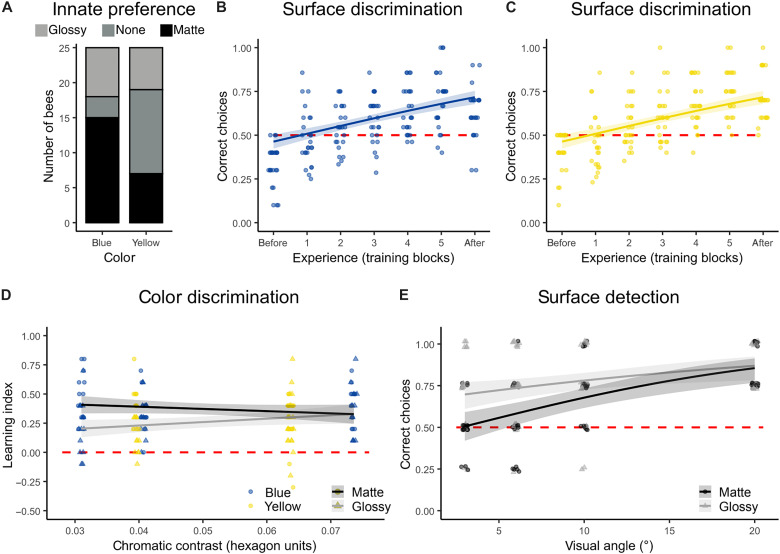
Behavioral responses of bumblebees to glossy and matte stimuli of different colors. (**A**) Bees show a color-dependent preference for matte stimuli, based on 10 approach flights (glossy: preference for glossy stimuli; matte: preference for matte stimuli; none: no preference). (**B** and **C**) Surface type can be used as a foraging cue for blue (B) and yellow stimuli (C; overlapping data points appear darker). (**D**) Fine color discrimination is more difficult when the stimuli are glossy than when they are matte. (**E**) Surface gloss enhances stimulus detection at smaller visual angles, based on four approach flights per visual angle.

Bees exhibit an innate color-dependent preference for matte stimuli but can overcome their preference through learning. Naïve bees were confronted with three glossy and three matte unrewarding stimuli of the same color (*n* = 25 bees per color), and their first 10 responses were used to quantify innate preference. For blue stimuli, bees visited matte stimuli significantly more often than glossy stimuli (*z* = −2.392, *P* = 0.017). By contrast, naïve bees had no preference for either surface type when the stimulus was yellow. We attribute this difference in innate preference to the fact that glossy surface reflections are better visible on dark backgrounds (Figs. 1 to 3) (*33*, *34*), because scattering by the interior is minimal for darkly pigmented areas. Over the course of the learning experiment, an increasing frequency of visits to nonpreferred stimuli offering a sucrose reward revealed that bees learned to use the type of surface reflection (glossy or matte) to discriminate between rewarding and unrewarding stimuli. After training, bumblebees visited their nonpreferred stimulus type more than before (Fig. 4, B and C; *n* = 25 bees per color). The fitted binomial logistic model of learning provided a significantly better fit than a null model for both blue (*z* = 3.2, *P* = 0.001) and yellow (*z* = 7.3, *P* < 0.001), supporting the conclusion that bees can use the degree of surface gloss as a foraging cue regardless of stimulus color.

To investigate the impact of surface gloss on object discriminability, we tested individual bee learning capability to distinguish two colors of varying similarity that were either glossy or matte. After training, bees were tested to discriminate between the same two colors of the same surface type as during the test for innate preference (*n* = 26 to 38 bees per color pair). We calculated a learning index that quantifies how much a bee improved, considering the strength of its innate preference (*35*). Glossy stimuli are significantly less discriminable than matte stimuli (χ = 10.8, *P* = 0.001), and this effect is strongest when chromatic contrast is low, as revealed by a significant interactive effect of chromatic contrast and surface type in the linear mixed model (χ = 5.3, *P* = 0.021). When visual contrast between the stimuli is high, bees can discriminate the two stimuli regardless of the surface type but with low chromatic contrast; however, the learning indices are significantly lower for glossy than for matte stimuli (Fig. 4D). The reduction in discriminability of colors caused by surface gloss is independent of pigment color, as learning indices for glossy yellow and glossy blue stimuli are similarly low.

The effect of surface gloss on long-range detection was tested in a Y-maze, by systematically moving the target away from the bee’s decision point to the point that was at the lower limit of the bumblebee visual detection threshold (*36*) (*n* = 19 bees per surface type). Fitting a generalized linear mixed model to test for the effect of surface type on detection while considering individual bee performance reveals that surface gloss significantly enhances object detection (χ = 8.8, *P* = 0.003). At large subtended visual angles, i.e., short distances, behavioral responses to matte and glossy stimuli converge, meaning that surface type has no effect on the likelihood of detection (Fig. 4E). By contrast, surface gloss increases detection at large distances. Surface gloss significantly increases detectability of stimuli at visual angles of 6° and 3° (Fig. 4E), which is at the threshold for visual detection by bumblebees (*36*). Bright reflections emitted by glossy surfaces can thus make flowers visible from distances at which they would be inconspicuous without gloss.

## DISCUSSION

Dynamic visual effects such as glossiness are taxonomically widespread, although their importance for visual signaling is poorly understood. The repeated occurrence of glossy visual effects in unrelated plant and animal signaling structures suggests that glossiness aids visibility under specific circumstances. Using ecologically relevant training conditions and realistic artificial stimuli, we reveal that object glossiness constitutes a trade-off for visual signaling. Surface gloss makes flowers detectable from distances at which matte flowers of the same size and color are undetectable. The compromised object discriminability likely entails an ecological cost of being glossy, for both plants and pollinators. Impeded color discrimination of co-occurring flowers by bees reduces flower constancy (*37*, *38*) and so increases interspecific pollen transfer—a major fitness cost for plants (*39*)—and decreases bee foraging efficiency (*31*). Enhanced detectability may explain why glossiness has evolved repeatedly across the Angiosperm phylogeny. Dynamic visual displays in animals entail active movement. The typical zigzag flight pattern of foraging insects (*40*) and limited movement of flowers by wind further enhance the visual dynamicity during a pollinator’s approach of a glossy flower. Floral gloss is particularly visible in the full sun, so glossy visual effects are stronger in a moving flower in the field than in the static condition of our arena.

Our study answers the long-held question of why so many flowers have conical epidermal cells (*17*, *18*), but the visual ecological implications of our study span well beyond plant-pollinator signaling and to different interaction types. Plant epidermal cell shape can be important for abiotic processes, such as mechanical strength or temperature regulation (*17*, *20*); here, we demonstrate how floral epidermal cell shape plays a key role for visual signaling. Plant surfaces, such as leaves, stems, and abaxial floral sides, are by default flat, but most Angiosperm flowers have conical adaxial epidermal cells. Previous modeling studies suggested that conical epidermal cells are “micro lenses” that focus incident light on the floral pigment (*23*, *41*); however, these studies considered only ideal, perpendicular illumination and did not incorporate natural variation and disorder that characterizes floral epidermal cell layers (*18*, *42*–*44*). Behavioral experiments with bumblebees provided inconclusive results on the behavioral relevance of any “lensing” effect (*45*). We propose the alternative explanation that cone-shaped epidermal cells enhance the spatial consistency of the visual signal, akin to corneal nipples on moth eyes (*46*) and diffusely reflecting microstructures in snake skin and butterfly wings (*47*, *48*). Diffuse reflection thus improves the discriminability and recognition of flowers by bees—with corresponding implications for signaling in animals. The reduced recognition caused by surface gloss is relevant for other types of visual communication, such as predator-prey interactions. Surface gloss hinders the ability to track moving prey by praying mantids and jumping spiders (*11*, *13*), and flash effects reduce the likelihood of bird pecks hitting a target (*12*, *49*). Glossy reflections can furthermore be a cue to locate water (*16*) or a female insect mate, which can be mimicked by sexually deceptive plants to attract male pollinating insects (*8*).

An intriguing open question is what visual channels are involved in processing glossy visual effects. The light reflected by glossy objects is polarized, although polarization effects are not processed by the part of the bee eye that viewed the stimuli (*50*), so polarization is unlikely to play a role in foraging (*43*). Both the chromatic and/or the achromatic visual pathway may be involved in processing glossy visual effects, but their relative importance remains unclear (fig. S1). Bees typically use chromatic cues for short distance visual tasks (*30*, *31*), such as those in the discrimination experiments, although achromatic information can feed into the chromatic visual pathway in a suite of insects (*51*–*54*). The effects of glossiness on discrimination and detection may be caused by the temporal variability of the stimuli, the extreme brightness emitted by glossy objects, or both. Bees can learn to use the temporal components of a visual cue while foraging (*55*). Achromatic information may be fed directly into bee motor systems, triggering rapid responses (*56*).

We conclude that glossy visual effects bear a visual ecological trade-off. Flat and smooth surfaces occur in floral signaling structures of unrelated clades, suggesting that species-specific conditions provide a selective advantage of flower gloss. Bright, directional reflections of glossy flowers enhance long-range detection but compromise object discriminability. Our results provide a functional explanation to the fast-growing literature on the role of dynamic coloration for attraction and avoidance.

## MATERIALS AND METHODS

### Spectroscopy

Reflectance spectra of intact flowers and artificial stimuli were recorded with an integrating sphere (Avasphere-50, Avantes, Apeldoorn, the Netherlands) using a Deuterium-Halogen lamp [Avantes, AvaLight D(H)-S] and a white reflectance standard (WS-2, Avantes) as a reference. In addition, reflectance spectra of intact flowers and artificial stimuli were measured as a function of angle of light incidence in a goniometric setup with two rotatable, coplanar optical fibers. One fiber delivers light from a halogen-deuterium light source [AvaLightD(H)-S], and the second fiber is connected to an AvaSpec-2048 spectrometer. The angular resolution of the setup has a Gaussian shape with half-width of ~5° (*25*). Reflectance spectra were measured by varying the illumination angle from −70° to 70° (−50° to 50° for small flowers of *Ranunculus*) in steps of 10°, recording the spectra at each illumination position in steps of 10°.

### Stimuli development

The artificial stimuli used for the behavioral trials were composed of epoxy mixed with pigments of different colors. We used matte petals of *C. bipinnatus* and glossy leaves of *Zamioculcas zamiifolia* as templates, because these structures provided large enough surfaces to stamp out circular stimuli (Ø 3.4 cm). Impressions of cone and flat epidermal cells were obtained using dental impression material (Provil novo, Kulzer GmbH, Hanau, Germany) (*19*). The negative mold was filled with pigmented epoxy (Epoxywinkel B.V., Diemen, the Netherlands). We used ultramarine blue (RAL5002), ocean blue (RAL5020), sky blue (RAL5015), pastel blue (RAL5024), azure blue (RAL5009), rapeseed yellow (RAL1021), sun yellow (RAL1037), and signal yellow (RAL1003). The resulting hardened epoxy yielded a facsimile of flower surfaces with different colors.

### Behavioral experiments

The experiments were conducted with *B. terrestris* colonies, obtained from Biobest (Westero, Belgium). The colonies were housed in a wooden two-chamber nesting box (43 cm by 23 cm by 11 cm), with one chamber for nest building and one for feeding. The bees were fed with APIinvert (Südzucker, Germany) and pollen (Bio-Blütenpollen, Naturwaren-Niederrhein, Germany), before testing. The stimuli were always presented vertically attached to plastic hangers (6.25 cm by 9.5 cm), covered in gray cardboard (Mi-Teintes #122, Canson SAS, Annonay Cedex, France) and a small balcony at the bottom edge on which a small feeder was placed.

Preference, learning, and discrimination experiments were conducted in a wooden flight arena (fig. S2). The arena measured 125 cm by 75 cm by 62 cm with a wooden turntable (Ø 56 cm) attached to the back wall of the arena. The arena was covered with a transparent acrylic lid and connected to the nesting box via an acrylic tube. The floor of the arena and the turntable were covered with gray cardboard. A focused light beam provided by a light guide with a collimating lens attached to a light-emitting diode (LED) light source (CL 6000, ZEISS, Photonic GmbH & CoKG, Vienna) illuminated the turntable and back wall in both experiments.

Bumblebee foragers were selected by observing their individual foraging activity on gray training stimuli, supplied with 1 M sucrose solution. Selected foragers were marked with number tags. Bees were tested individually to exclude social learning as a factor. Before testing, each bee first performed a pretraining, consisting of three foraging bouts from the training stimuli, rewarded with 10 μl of 1 M sucrose solution, to accustom it to the experimental conditions. Feeders were refilled immediately after a visit. To test for preferences, the bees were presented with three matte and three glossy stimuli of the same color (either ultramarine blue or rapeseed yellow) attached to hangers with empty feeders and allowed to choose 10 times. A choice was counted when the bee touched the feeder. After 10 (unrewarded) choices, the bee was allowed to feed ad libitum from 1 M sucrose solution, before returning to the colony. Significance was tested using a generalized linear mixed model, using the choices for matte versus glossy stimuli as the response variable, color as the fixed effect, and bee ID as the random effect.

In the subsequent training, bees were presented three rewarded and three unrewarded stimuli, with the bee’s nonpreferred stimulus as the rewarding stimulus type. If bees had chosen fifty-fifty in the innate preference test, we flipped a coin to assign them to a training protocol. Bees were allowed to forage until achieving 30 successful visits to the rewarding stimulus. After the last visit, bees were allowed to feed ad libitum from a neutral stimulus, to ensure that all bees perceived the same learning events. To test the bees’ learning success, an unrewarded final test was performed, identical to the preference test, i.e., 10 unrewarded choices were recorded for each bee. We tested for significance using a generalized linear mixed model with the correct versus incorrect choices as response variables, training block as the fixed effect, and bee ID as the random effect.

During the discrimination experiments, bees were presented with stimuli pairs of different colors, i.e., ultramarine blue and ocean blue, sky blue and azure blue, sky blue and pastel blue, sun yellow and rapeseed yellow, or sun yellow and signal yellow. Details about the used colors are provided in fig. S3. Each color pair was tested for matte and glossy, which translates to 10 combinations total. A learning index (*35*) was calculated by subtracting the proportion of correct (i.e., nonpreferred) choices in the preference test from the proportion of correct choices of the final test. Theoretically, the learning index can vary between 1 and −0.5, whereas a value larger than 0 indicates that the bee has learned to choose the rewarding stimulus, and smaller than 0 indicates that the bee chose the unrewarded (and initially preferred) stimulus more often. Statistical significance was tested using a linear mixed model with the learning index as the response variable and an interaction between surface type and within-pair color contrast and a random effect of bee ID.

For the detection experiment, a flight arena (125 cm by 75 cm by 39 cm) was connected to a wooden Y-maze setup via a short acrylic tube (fig. S2) (*36*). The arena and the Y-maze were covered with a transparent acrylic lid, and walls and floor were covered with gray cardboard (Canson #122). The LED light source and a bifurcated light guide with collimating lenses were used to illuminate the back walls. All stimuli were made of ultramarine blue (RAL5002). In addition, a smaller set of 1.1 cm in diameter stimuli was created by stamping out existing stimuli to allow the testing of small visual angles (see below).

During the experiment, bees were presented with a rewarding and unrewarding platform, attached to a stand in each of the Y-maze arms. The rewarding platform offered 200 μl of 1.5 M sucrose solution and a stimulus, and the unrewarded platform contained 200 μl of water. The stimulus was presented at visual angles of 20, 10, 6, and 3 and pseudorandomly exchanged between the two arms, i.e., randomized allocation but never more than three consecutive turns in the same arm to avoid bees developing a side preference (*57*). A decision was recorded as soon as the bee flew into a Y-maze arm. Bees started at a visual angle of 20° and were allowed to forage until four of five decisions were correct. After reaching this criterion, the bees made four choices per tested visual angle, in a decreasing order (*57*). We used a generalized linear mixed model to test for significance of correct versus incorrect choices (response variable) as a function of subtended visual angle and surface type while accounting for bee ID (random effect).

## Supplementary Materials

### This PDF file includes:

Figs. S1 to S3 References

## References

[1] D. J. Kemp, J. M. Macedonia, Structural ultraviolet ornamentation in the butterfly *Hypolimnas bolina* L. (Nymphalidae): Visual, morphological and ecological properties. Aust. J. Zool. 54, 235 (2006).

[2] D. Stuart-Fox, L. Ospina-Rozo, L. Ng, A. M. Franklin, The paradox of iridescent signals. Trends Ecol. Evol. 36, 187–195 (2021). 33168152 10.1016/j.tree.2020.10.009

[3] A. M. Franklin, L. Ospina-Rozo, Gloss. Curr. Biol. 31, R172–R173 (2020). 10.1016/j.cub.2020.11.065 33621499

[4] K. Lunau, Z.-X. Ren, X.-Q. Fan, J. Trunschke, G. H. Pyke, H. Wang, Nectar mimicry: A new phenomenon. Sci. Rep. 10, 7039 (2020). 32341437 10.1038/s41598-020-63997-3 PMC7184725

[5] J. Sosa Espinosa, D. G. Stavenga, C. J. van der Kooi, M. A. Giraldo, *Morpho* butterfly flashiness crucially depends on wing scale curvature. Biol. Lett. 20, 20240358 (2024). 39532147 10.1098/rsbl.2024.0358 PMC11557224

[6] C. J. van der Kooi, J. Spaethe, Visual ecology: How glossy colours shine and confuse. Curr. Biol. 33, R865–R867 (2023). 37607483 10.1016/j.cub.2023.07.011

[7] D. Osorio, A. D. Ham, Spectral reflectance and directional properties of structural coloration in bird plumage. J. Exp. Biol. 205, 2017–2027 (2002). 12089207 10.1242/jeb.205.14.2017

[8] M. L. de Jager, A. G. Ellis, Gender-specific pollinator preference for floral traits. Funct. Ecol. 26, 1197–1204 (2012).

[9] J. A. Endler, Signals, signal conditions, and the direction of evolution. Am. Nat. 139, S125–S153 (1992).

[10] S. A. Echeverri, A. E. Miller, J. Chen, E. W. McQueen, M. Plakke, M. Spicer, K. L. Hoke, M. C. Stoddard, N. I. Morehouse, How signaling geometry shapes the efficacy and evolution of animal communication systems. Integr. Comp. Biol. 61, 787–813 (2021). 34021338 10.1093/icb/icab090

[11] P. Henríquez-Piskulich, D. Stuart-Fox, M. Elgar, I. Marusic, A. M. Franklin, Dazzled by shine: Gloss as an antipredator strategy in fast moving prey. Behav. Ecol. 34, 862–871 (2023). 37744168 10.1093/beheco/arad046 PMC10516678

[12] S. Silvasti, D. J. Kemp, T. E. White, O. Nokelainen, J. Valkonen, J. Mappes, The flashy escape: Support for dynamic flash coloration as anti-predator defence. Biol. Lett. 20, 20240303 (2024). 39079677 10.1098/rsbl.2024.0303 PMC11288678

[13] A. M. Franklin, M. R. Brown, N. J. Willmott, Glossiness disrupts predator localisation of moving prey. Curr. Biol. 34, R1131–R1132 (2024). 39561703 10.1016/j.cub.2024.09.066

[14] S. Wilmsen, A. G. Dyer, K. Lunau, Conical flower cells reduce surface gloss and improve colour signal integrity for free-flying bumblebees. J. Pollinat. Ecol. 28, 108–126 (2021).

[15] A. M. Vallet, J. A. Coles, The perception of small objects by the drone honeybee. J. Comp. Physiol. A 172, 183–188 (1993).

[16] T. F. Mathejczyk, É. J. Babo, E. Schönlein, N. V. Grinda, A. Greiner, N. Okrožnik, G. Belušič, M. F. Wernet, Behavioral responses of free-flying *Drosophila melanogaster* to shiny, reflecting surfaces. J. Comp. Physiol. A 209, 929–941 (2023). 10.1007/s00359-023-01676-0 PMC10643280 37796303

[17] H. M. Whitney, K. M. V. Bennett, M. Dorling, L. Sandbach, D. Prince, L. Chittka, B. J. Glover, Why do so many petals have conical epidermal cells? Ann. Bot. 108, 609–616 (2011). 21470973 10.1093/aob/mcr065 PMC3170151

[18] Q. O. N. Kay, H. S. Daoud, C. H. Stirton, Pigment distribution, light reflection and cell structure in petals. Bot. J. Linn. Soc. 83, 57–83 (1981).

[19] M. Kraaij, C. J. van der Kooi, Surprising absence of association between flower surface microstructure and pollination system. Plant Biol. 22, 177–183 (2020). 31710761 10.1111/plb.13071 PMC7064994

[20] C. J. van der Kooi, J. T. M. Elzenga, J. Dijksterhuis, D. G. Stavenga, Functional optics of glossy buttercup flowers. J. R. Soc. Interface 17, 20160933 (2017). 10.1098/rsif.2016.0933 PMC5332578 28228540

[21] H. M. Whitney, S. A. Rands, N. J. Elton, A. G. Ellis, A technique for measuring petal gloss, with examples from the Namaqualand flora. PLOS ONE 7, e29476 (2012). 22253729 10.1371/journal.pone.0029476 PMC3254604

[22] S. Papiorek, R. R. Junker, K. Lunau, Gloss, colour and grip: Multifunctional epidermal cell shapes in bee- and bird-pollinated flowers. PLOS ONE 9, e112013 (2014). 25369510 10.1371/journal.pone.0112013 PMC4219824

[23] H. L. Gorton, T. C. Vogelmann, Effects of epidermal cell shape and pigmentation on optical properties of *Antirrhinum* petals at visible and ultraviolet wavelengths. Plant Physiol. 112, 879–888 (1996). 12226425 10.1104/pp.112.3.879 PMC158014

[24] C. Buschhaus, D. Hager, R. Jetter, Wax layers on *Cosmos bipinnatus* petals contribute unequally to total petal water resistance. Plant Physiol. 167, 80–88 (2014). 25413359 10.1104/pp.114.249235 PMC4281003

[25] D. G. Stavenga, M. Staal, C. J. van der Kooi, Conical epidermal cells cause velvety colouration and enhanced patterning in *Mandevilla* flowers. Faraday Discuss. 223, 98–106 (2020). 32719835 10.1039/d0fd00055h

[26] T. C. Vogelmann, Plant tissue optics. Annu. Rev. Plant Biol. 44, 231–251 (1993).

[27] E. Bukhanov, Y. Gurevich, M. Krakhalev, D. Shabanov, “Modeling optical properties of plant epicuticular wax” in *2020 International Conference on Information Technology and Nanotechnology (ITNT)* (IEEE, 2020), pp. 1–7.

[28] L. De Paola, T. A. Veldhuis, M. Kraaij, D. G. Stavenga, K. J. Tiedge, C. J. van der Kooi, Stacked scattering: The key to bright flowers lies in the mesophyll. Am. J. Bot. 112, e70104 (2025). 10.1002/ajb2.70104 PMC12816439 40996620

[29] L. Chittka, The colour hexagon: A chromaticity diagram based on photoreceptor excitations as a generalized representation of colour opponency. J. Comp. Physiol. A 170, 533–543 (1992).

[30] M. Giurfa, M. Vorobyev, P. Kevan, R. Menzel, Detection of coloured stimuli by honeybees: Minimum visual angles and receptor specific contrasts. J. Comp. Physiol. A 178, 699–709 (1996).

[31] J. Spaethe, J. Tautz, L. Chittka, Visual constraints in foraging bumblebees: Flower size and color affect search time and flight behavior. Proc. Natl. Acad. Sci. U.S.A. 98, 3898–3903 (2001). 11259668 10.1073/pnas.071053098 PMC31150

[32] A. Kelber, M. Vorobyev, D. Osorio, Animal colour vision–behavioural tests and physiological concepts. Biol. Rev. 78, 81–118 (2003). 12620062 10.1017/s1464793102005985

[33] S. Yoshioka, S. Kinoshita, Structural or pigmentary? Origin of the distinctive white stripe on the blue wing of a *Morpho* butterfly. Proc. R. Soc. B Biol. Sci. 273, 129–134 (2005). 10.1098/rspb.2005.3314 PMC1560023 16555778

[34] C. J. van der Kooi, A. G. Dyer, D. G. Stavenga, Is floral iridescence a biologically relevant cue in plant-pollinator signaling? New Phytol. 205, 18–20 (2015). 25243861 10.1111/nph.13066

[35] W. G. Quinn, W. A. Harris, S. Benzer, Conditioned behavior in *Drosophila melanogaster*. Proc. Natl. Acad. Sci. U.S.A. 71, 708–712 (1974). 4207071 10.1073/pnas.71.3.708 PMC388082

[36] A. G. Dyer, J. Spaethe, S. Prack, Comparative psychophysics of bumblebee and honeybee colour discrimination and object detection. J. Comp. Physiol. A 194, 617–627 (2008). 10.1007/s00359-008-0335-1 18437390

[37] L. Chittka, J. Spaethe, A. Schmidt, A. Hickelsberger “Adaptation, constraint, and chance in the evolution of flower color and pollinator color vision” in *Cognitive Ecology of Pollination: Animal Behavior and Floral Evolution* (Cambridge Univ. Press, 2001), pp. 106–126.

[38] A. G. Dyer, L. Chittka, Biological significance of distinguishing between similar colours in spectrally variable illumination: Bumblebees (*Bombus terrestris*) as a case study. J. Comp. Physiol. A 190, 105–114 (2004). 10.1007/s00359-003-0475-2 14652688

[39] C. Minnaar, B. Anderson, M. L. de Jager, J. D. Karron, Plant–pollinator interactions along the pathway to paternity. Ann. Bot. 123, 225–245 (2019). 30535041 10.1093/aob/mcy167 PMC6344347

[40] M. Streinzer, H. F. Paulus, J. Spaethe, Floral colour signal increases short-range detectability of a sexually deceptive orchid to its bee pollinator. J. Exp. Biol. 212, 1365–1370 (2009). 19376957 10.1242/jeb.027482

[41] B. D. Wilts, P. J. Rudall, E. Moyroud, T. Gregory, Y. Ogawa, S. Vignolini, U. Steiner, B. J. Glover, Ultrastructure and optics of the prism-like petal epidermal cells of *Eschscholzia californica* (California poppy). New Phytol. 219, 1124–1133 (2018). 29856474 10.1111/nph.15229 PMC6055853

[42] D. W. Lee, *Nature’s Palette. The Science of Plant Color* (University of Chicago Press, 2007).

[43] C. J. van der Kooi, A. G. Dyer, P. G. Kevan, K. Lunau, Functional significance of the optical properties of flowers for visual signalling. Ann. Bot. 123, 263–276 (2019). 29982325 10.1093/aob/mcy119 PMC6344213

[44] B. Fritz, R. Hünig, R. Schmager, M. Hetterich, U. Lemmer, G. Gomard, Assessing the influence of structural disorder on the plant epidermal cells’ optical properties: A numerical analysis. Bioinspir. Biomim. 12, 036011 (2017). 28471745 10.1088/1748-3190/aa6c46

[45] A. G. Dyer, H. M. Whitney, S. E. J. Arnold, B. J. Glover, L. Chittka, Mutations perturbing petal cell shape and anthocyanin synthesis influence bumblebee perception of *Antirrhinum majus* flower colour. Arthropod Plant Interact. 1, 45–55 (2007).

[46] D. G. Stavenga, S. Foletti, G. Palasantzas, K. Arikawa, Light on the moth-eye corneal nipple array of butterflies. Proc. R. Soc. B Biol. Sci. 273, 661–667 (2006). 10.1098/rspb.2005.3369 PMC1560070 16608684

[47] M. Spinner, A. Kovalev, S. N. Gorb, G. Westhoff, Snake velvet black: Hierarchical micro- and nanostructure enhances dark colouration in *Bitis rhinoceros*. Sci. Rep. 3, 1846 (2013). 23677278 10.1038/srep01846 PMC3655483

[48] D. G. Stavenga, S. Stowe, K. Siebke, J. Zeil, K. Arikawa, Butterfly wing colours: Scale beads make white pierid wings brighter. Proc. R. Soc. B 271, 1577–1584 (2004). 10.1098/rspb.2004.2781 PMC1691762 15306303

[49] T. W. Pike, Interference coloration as an anti-predator defence. Biol. Lett. 11, 20150159 (2015). 25878050 10.1098/rsbl.2015.0159 PMC4424625

[50] R. Wehner, G. D. Bernard, E. Geiger, Twisted and non-twisted rhabdoms and their significance for polarization detection in the bee. J. Comp. Physiol. A 104, 225–245 (1975).

[51] M. Pagni, V. Haikala, V. Oberhauser, P. B. Meyer, D. F. Reiff, C. Schnaitmann, Interaction of “chromatic” and “achromatic” circuits in *Drosophila* color opponent processing. Curr. Biol. 31, 1687–1698.e4 (2021). 33636123 10.1016/j.cub.2021.01.105

[52] C. Schnaitmann, C. Garbers, T. Wachtler, H. Tanimoto, Color discrimination with broadband photoreceptors. Curr. Biol. 23, 2375–2382 (2013). 24268411 10.1016/j.cub.2013.10.037

[53] F. J. Stewart, M. Kinoshita, K. Arikawa, The butterfly *Papilio xuthus* detects visual motion using chromatic contrast. Biol. Lett. 11, 20150687 (2015). 26490417 10.1098/rsbl.2015.0687 PMC4650181

[54] C. J. van der Kooi, A. Kelber, Achromatic cues are important for flower visibility to hawkmoths and other insects. Front. Ecol. Evol. 10, 819436 (2022).

[55] L. Ng, L. Ospina-Rozo, J. E. Garcia, A. G. Dyer, D. Stuart-Fox, Iridescence untwined: Honey bees can separate hue variations in space and time. Behav. Ecol. 33, 884–891 (2022).

[56] P. Skorupski, L. Chittka, Differences in photoreceptor processing speed for chromatic and achromatic vision in the bumblebee, *Bombus terrestris*. J. Neurosci. 30, 3896–3903 (2010). 20237260 10.1523/JNEUROSCI.5700-09.2010 PMC6632265

[57] J. Spaethe, L. Chittka, Interindividual variation of eye optics and single object resolution in bumblebees. J. Exp. Biol. 206, 3447–3453 (2003). 12939375 10.1242/jeb.00570

[58] A. D. Briscoe, L. Chittka, The evolution of color vision in insects. Annu. Rev. Entomol. 46, 471–510 (2001). 11112177 10.1146/annurev.ento.46.1.471

[59] C. J. van der Kooi, D. G. Stavenga, G. Belusic, K. Arikawa, A. Kelber, Evolution of insect color vision: From spectral sensitivity to visual ecology. Annu. Rev. Entomol. 66, 435–461 (2021). 32966103 10.1146/annurev-ento-061720-071644

[60] T. Ausma, V. Bansal, M. Kraaij, A. C. M. Verloop, A. Gasperl, M. Müller, S. Kopriva, L. J. De Kok, C. J. van der Kooi, Floral displays suffer from sulphur deprivation. Environ. Exp. Bot. 192, 104656 (2021).

