# Supplementary material for: Dynamic visual effects enhance flower conspicuousness but compromise color perception
Source: Sci Adv. 2025 Nov 26;11(48):eadz9010. doi: 10.1126/sciadv.adz9010 (PMC12652320; doi:10.1126/sciadv.adz9010)
Supplement: Supplementary file 1 — Figs. S1 to S3 References [file sciadv.adz9010_sm.pdf]

Science Advances
AAAS

Supplementary Materials for

Dynamic visual effects enhance flower conspicuousness but compromise color perception

Alexander Dietz *et al*.

Corresponding author: Casper J. van der Kooi, c.j.van.der.kooi@rug.nl

Sci. Adv. **11**, eadz9010 (2025)
DOI: 10.1126/sciadv.adz9010

This PDF file includes:

Figs. S1 to S3
References

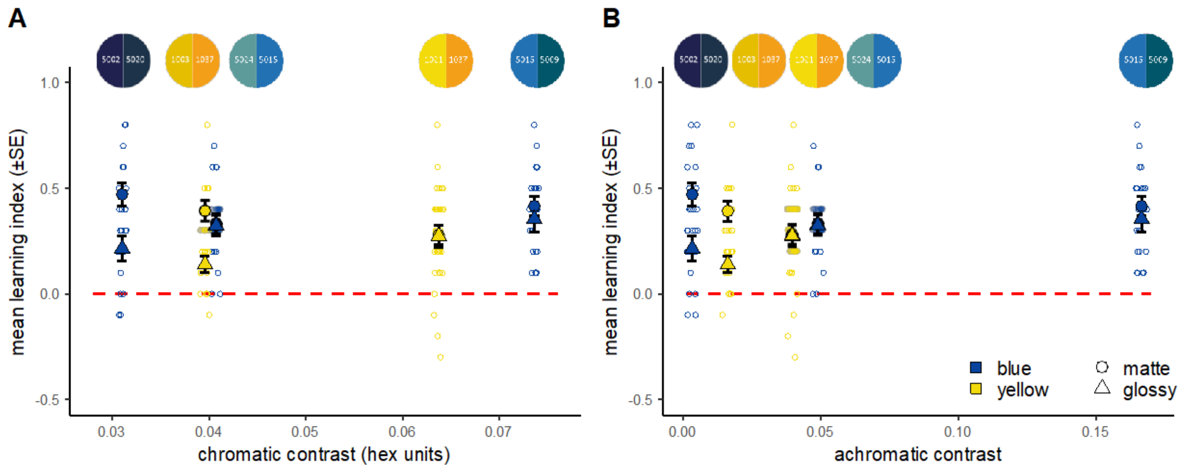

Figure S1. Colour discrimination for glossy and matte colour pairs of varying difference. A: Discrimination as a function of within-pair colour contrast (as in Fig. 4D). B: Discrimination as a function of within-pair achromatic (long-wavelength photoreceptor) contrast. Reflectance spectra of the stimuli are provided in Figure S3. Within-pair colour contrast was calculated with the colour hexagon (*29*), using a D65 illumination, bumblebee photoreceptor spectral sensitivities (*58*, *59*), and the Canson #122 background used in the behavioural experiment. Within-pair achromatic contrast was calculated as per Spaethe et al. (*31*).

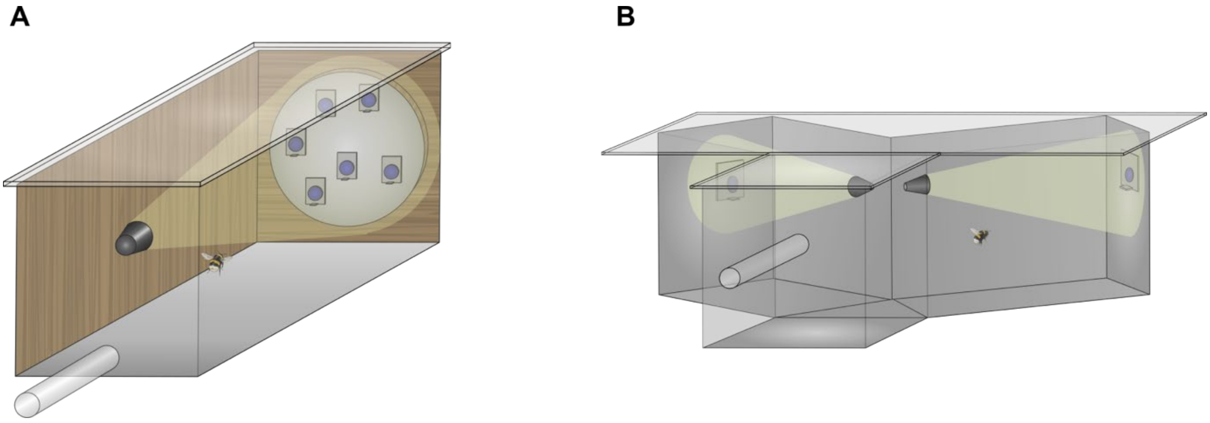

Figure S2. Visualisation of the experimental arenas used for the bumblebee behavioural experiments. A: Experimental setup used for the preference and colour discrimination tests. B: Y-maze used for the detection experiments.

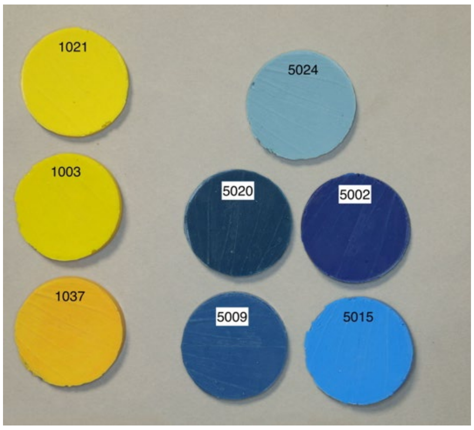

Figure S3. Colour stimuli and background used in the experiment. The left image shows a picture of the used stimuli with their RAL numbers on the used Canson #122 background. Middle and right: reflectance spectra of matte stimuli of different colours, obtained with a bifurcated probe *(60)*. Numbers next to the curves correspond to RAL numbers of different colours (see Fig. S1). The Canson #122 grey background spectrum is shown in black in the middle panel ("Back").

REFERENCES AND NOTES

1. D. J. Kemp, J. M. Macedonia, Structural ultraviolet ornamentation in the butterfly *Hypolimnas bolina* L. (Nymphalidae): Visual, morphological and ecological properties. *Aust. J. Zool.* **54**, 235 (2006).

2. D. Stuart-Fox, L. Ospina-Rozo, L. Ng, A. M. Franklin, The paradox of iridescent signals. *Trends Ecol. Evol.* **36**, 187–195 (2021).

3. A. M. Franklin, L. Ospina-Rozo, Gloss. *Curr. Biol.* **31**, R172–R173 (2020).

4. K. Lunau, Z.-X. Ren, X.-Q. Fan, J. Trunschke, G. H. Pyke, H. Wang, Nectar mimicry: A new phenomenon. *Sci. Rep.* **10**, 7039 (2020).

5. J. Sosa Espinosa, D. G. Stavenga, C. J. van der Kooi, M. A. Giraldo, *Morpho* butterfly flashiness crucially depends on wing scale curvature. *Biol. Lett.* **20**, 20240358 (2024).

6. C. J. van der Kooi, J. Spaethe, Visual ecology: How glossy colours shine and confuse. *Curr. Biol.* **33**, R865–R867 (2023).

7. D. Osorio, A. D. Ham, Spectral reflectance and directional properties of structural coloration in bird plumage. *J. Exp. Biol.* **205**, 2017–2027 (2002).

8. M. L. de Jager, A. G. Ellis, Gender-specific pollinator preference for floral traits. *Funct. Ecol.* **26**, 1197–1204 (2012).

9. J. A. Endler, Signals, signal conditions, and the direction of evolution. *Am. Nat.* **139**, S125–S153 (1992).

10. S. A. Echeverri, A. E. Miller, J. Chen, E. W. McQueen, M. Plakke, M. Spicer, K. L. Hoke, M. C. Stoddard, N. I. Morehouse, How signaling geometry shapes the efficacy and evolution of animal communication systems. *Integr. Comp. Biol.* **61**, 787–813 (2021).

11. P. Henríquez-Piskulich, D. Stuart-Fox, M. Elgar, I. Marusic, A. M. Franklin, Dazzled by shine: Gloss as an antipredator strategy in fast moving prey. *Behav. Ecol.* **34**, 862–871 (2023).

12. S. Silvasti, D. J. Kemp, T. E. White, O. Nokelainen, J. Valkonen, J. Mappes, The flashy escape: Support for dynamic flash coloration as anti-predator defence. *Biol. Lett.* **20**, 20240303 (2024).

13. A. M. Franklin, M. R. Brown, N. J. Willmott, Glossiness disrupts predator localisation of moving prey. *Curr. Biol.* **34**, R1131–R1132 (2024).

14. S. Wilmsen, A. G. Dyer, K. Lunau, Conical flower cells reduce surface gloss and improve colour signal integrity for free-flying bumblebees. *J. Pollinat. Ecol.* **28**, 108–126 (2021).

15. A. M. Vallet, J. A. Coles, The perception of small objects by the drone honeybee. *J. Comp. Physiol. A* **172**, 183–188 (1993).

16. T. F. Mathejczyk, É. J. Babo, E. Schönlein, N. V. Grinda, A. Greiner, N. Okrožnik, G. Belušič, M. F. Wernet, Behavioral responses of free-flying *Drosophila melanogaster* to shiny, reflecting surfaces. *J. Comp. Physiol. A* **209**, 929–941 (2023).

17. H. M. Whitney, K. M. V. Bennett, M. Dorling, L. Sandbach, D. Prince, L. Chittka, B. J. Glover, Why do so many petals have conical epidermal cells? *Ann. Bot.* **108**, 609–616 (2011).

18. Q. O. N. Kay, H. S. Daoud, C. H. Stirton, Pigment distribution, light reflection and cell structure in petals. *Bot. J. Linn. Soc.* **83**, 57–83 (1981).

19. M. Kraaij, C. J. van der Kooi, Surprising absence of association between flower surface microstructure and pollination system. *Plant Biol.* **22**, 177–183 (2020).

20. C. J. van der Kooi, J. T. M. Elzenga, J. Dijksterhuis, D. G. Stavenga, Functional optics of glossy buttercup flowers. *J. R. Soc. Interface* **17**, 20160933 (2017).

21. H. M. Whitney, S. A. Rands, N. J. Elton, A. G. Ellis, A technique for measuring petal gloss, with examples from the Namaqualand flora. *PLOS ONE* **7**, e29476 (2012).

22. S. Papiorek, R. R. Junker, K. Lunau, Gloss, colour and grip: Multifunctional epidermal cell shapes in bee- and bird-pollinated flowers. *PLOS ONE* **9**, e112013 (2014).

23. H. L. Gorton, T. C. Vogelmann, Effects of epidermal cell shape and pigmentation on optical properties of *Antirrhinum* petals at visible and ultraviolet wavelengths. *Plant Physiol.* **112**, 879–888 (1996).

24. C. Buschhaus, D. Hager, R. Jetter, Wax layers on *Cosmos bipinnatus* petals contribute unequally to total petal water resistance. *Plant Physiol.* **167**, 80–88 (2014).

25. D. G. Stavenga, M. Staal, C. J. van der Kooi, Conical epidermal cells cause velvety colouration and enhanced patterning in *Mandevilla* flowers. *Faraday Discuss.* **223**, 98–106 (2020).

26. T. C. Vogelmann, Plant tissue optics. *Annu. Rev. Plant Biol.* **44**, 231–251 (1993).

27. E. Bukhanov, Y. Gurevich, M. Krakhalev, D. Shabanov, "Modeling optical properties of plant epicuticular wax" in *2020 International Conference on Information Technology and Nanotechnology (ITNT)* (IEEE, 2020), pp. 1–7.

28. L. De Paola, T. A. Veldhuis, M. Kraaij, D. G. Stavenga, K. J. Tiedge, C. J. van der Kooi, Stacked scattering: The key to bright flowers lies in the mesophyll. *Am. J. Bot.* **112**, e70104 (2025).

29. L. Chittka, The colour hexagon: A chromaticity diagram based on photoreceptor excitations as a generalized representation of colour opponency. *J. Comp. Physiol. A* **170**, 533–543 (1992).

30. M. Giurfa, M. Vorobyev, P. Kevan, R. Menzel, Detection of coloured stimuli by honeybees: Minimum visual angles and receptor specific contrasts. *J. Comp. Physiol. A* **178**, 699–709 (1996).

31. J. Spaethe, J. Tautz, L. Chittka, Visual constraints in foraging bumblebees: Flower size and color affect search time and flight behavior. *Proc. Natl. Acad. Sci. U.S.A.* **98**, 3898–3903 (2001).

32. A. Kelber, M. Vorobyev, D. Osorio, Animal colour vision–behavioural tests and physiological concepts. *Biol. Rev.* **78**, 81–118 (2003).

33. S. Yoshioka, S. Kinoshita, Structural or pigmentary? Origin of the distinctive white stripe on the blue wing of a *Morpho* butterfly. *Proc. R. Soc. B Biol. Sci.* **273**, 129–134 (2005).

34. C. J. van der Kooi, A. G. Dyer, D. G. Stavenga, Is floral iridescence a biologically relevant cue in plant-pollinator signaling? *New Phytol.* **205**, 18–20 (2015).

35. W. G. Quinn, W. A. Harris, S. Benzer, Conditioned behavior in *Drosophila melanogaster*. *Proc. Natl. Acad. Sci. U.S.A.* **71**, 708–712 (1974).

36. A. G. Dyer, J. Spaethe, S. Prack, Comparative psychophysics of bumblebee and honeybee colour discrimination and object detection. *J. Comp. Physiol. A* **194**, 617–627 (2008).

37. L. Chittka, J. Spaethe, A. Schmidt, A. Hickelsberger "Adaptation, constraint, and chance in the evolution of flower color and pollinator color vision" in *Cognitive Ecology of Pollination: Animal Behavior and Floral Evolution* (Cambridge Univ. Press., 2001), pp. 106–126.

38. A. G. Dyer, L. Chittka, Biological significance of distinguishing between similar colours in spectrally variable illumination: Bumblebees (*Bombus terrestris*) as a case study. *J. Comp. Physiol. A* **190**, 105–114 (2004).

39. C. Minnaar, B. Anderson, M. L. de Jager, J. D. Karron, Plant–pollinator interactions along the pathway to paternity. *Ann. Bot.* **123**, 225–245 (2019).

40. M. Streinzer, H. F. Paulus, J. Spaethe, Floral colour signal increases short-range detectability of a sexually deceptive orchid to its bee pollinator. *J. Exp. Biol.* **212**, 1365–1370 (2009).

41. B. D. Wilts, P. J. Rudall, E. Moyroud, T. Gregory, Y. Ogawa, S. Vignolini, U. Steiner, B. J. Glover, Ultrastructure and optics of the prism-like petal epidermal cells of *Eschscholzia californica* (California poppy). *New Phytol.* **219**, 1124–1133 (2018).

42. D. W. Lee, *Nature's Palette. The Science of Plant Color* (University of Chicago Press, 2007).

43. C. J. van der Kooi, A. G. Dyer, P. G. Kevan, K. Lunau, Functional significance of the optical properties of flowers for visual signalling. *Ann. Bot.* **123**, 263–276 (2019).

44. B. Fritz, R. Hünig, R. Schmager, M. Hetterich, U. Lemmer, G. Gomard, Assessing the influence of structural disorder on the plant epidermal cells' optical properties: A numerical analysis. *Bioinspir. Biomim.* **12**, 036011 (2017).

45. A. G. Dyer, H. M. Whitney, S. E. J. Arnold, B. J. Glover, L. Chittka, Mutations perturbing petal cell shape and anthocyanin synthesis influence bumblebee perception of *Antirrhinum majus* flower colour. *Arthropod Plant Interact.* **1**, 45–55 (2007).

46. D. G. Stavenga, S. Foletti, G. Palasantzas, K. Arikawa, Light on the moth-eye corneal nipple array of butterflies. *Proc. R. Soc. B Biol. Sci.* **273**, 661–667 (2006).

47. M. Spinner, A. Kovalev, S. N. Gorb, G. Westhoff, Snake velvet black: Hierarchical micro- and nanostructure enhances dark colouration in *Bitis rhinoceros*. *Sci. Rep.* **3**, 1846 (2013).

48. D. G. Stavenga, S. Stowe, K. Siebke, J. Zeil, K. Arikawa, Butterfly wing colours: Scale beads make white pierid wings brighter. *Proc. R. Soc. B* **271**, 1577–1584 (2004).

49. T. W. Pike, Interference coloration as an anti-predator defence. *Biol. Lett.* **11**, 20150159 (2015).

50. R. Wehner, G. D. Bernard, E. Geiger, Twisted and non-twisted rhabdoms and their significance for polarization detection in the bee. *J. Comp. Physiol. A* **104**, 225–245 (1975).

51. M. Pagni, V. Haikala, V. Oberhauser, P. B. Meyer, D. F. Reiff, C. Schnaitmann, Interaction of "chromatic" and "achromatic" circuits in *Drosophila* color opponent processing. *Curr. Biol.* **31**, 1687–1698.e4 (2021).

52. C. Schnaitmann, C. Garbers, T. Wachtler, H. Tanimoto, Color discrimination with broadband photoreceptors. *Curr. Biol.* **23**, 2375–2382 (2013).

53. F. J. Stewart, M. Kinoshita, K. Arikawa, The butterfly *Papilio xuthus* detects visual motion using chromatic contrast. *Biol. Lett.* **11**, 20150687 (2015).

54. C. J. van der Kooi, A. Kelber, Achromatic cues are important for flower visibility to hawkmoths and other insects. *Front. Ecol. Evol.* **10**, 819436 (2022).

55. L. Ng, L. Ospina-Rozo, J. E. Garcia, A. G. Dyer, D. Stuart-Fox, Iridescence untwined: Honey bees can separate hue variations in space and time. *Behav. Ecol.* **33**, 884–891 (2022).

56. P. Skorupski, L. Chittka, Differences in photoreceptor processing speed for chromatic and achromatic vision in the bumblebee, *Bombus terrestris*. *J. Neurosci.* **30**, 3896–3903 (2010).

57. J. Spaethe, L. Chittka, Interindividual variation of eye optics and single object resolution in bumblebees. *J. Exp. Biol.* **206**, 3447–3453 (2003).

58. A. D. Briscoe, L. Chittka, The evolution of color vision in insects. *Annu. Rev. Entomol.* **46**, 471–510 (2001).

59. C. J. van der Kooi, D. G. Stavenga, G. Belusic, K. Arikawa, A. Kelber, Evolution of insect color vision: From spectral sensitivity to visual ecology. *Annu. Rev. Entomol.* **66**, 435–461 (2021).

60. T. Ausma, V. Bansal, M. Kraaij, A. C. M. Verloop, A. Gasperl, M. Müller, S. Kopriva, L. J. De Kok, C. J. van der Kooi, Floral displays suffer from sulphur deprivation. *Environ. Exp. Bot.* **192**, 104656 (2021).
